# Supplementary material for: Identification of photoperiod-induced specific miRNAs in the adrenal glands of Sunite sheep (Ovis aries)
Source: Front Vet Sci. 2022 Jul 22;9:888207. doi: 10.3389/fvets.2022.888207 (PMC9354845; doi:10.3389/fvets.2022.888207)
Supplement: Supplementary file 1 [file Data_Sheet_1.ZIP › Supplementary file/miRDeep2 file.zip!/miRDeep2 file/Novel_Structure/LP42A1/Novel_463.pdf]

Provisional ID : NC_019470.2_21747
Score total : 1.9
Score for star read(s) : -1.3
Score for read counts : 0
Score for mfe : 1.6
Score for randfold : 1.6
Score for cons. seed :
Total read count : 30
Mature read count : 30
Loop read count : 0
Star read count : 0

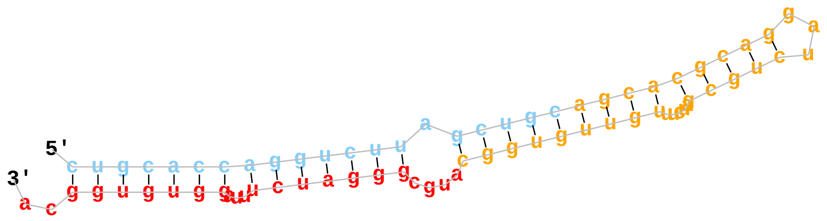

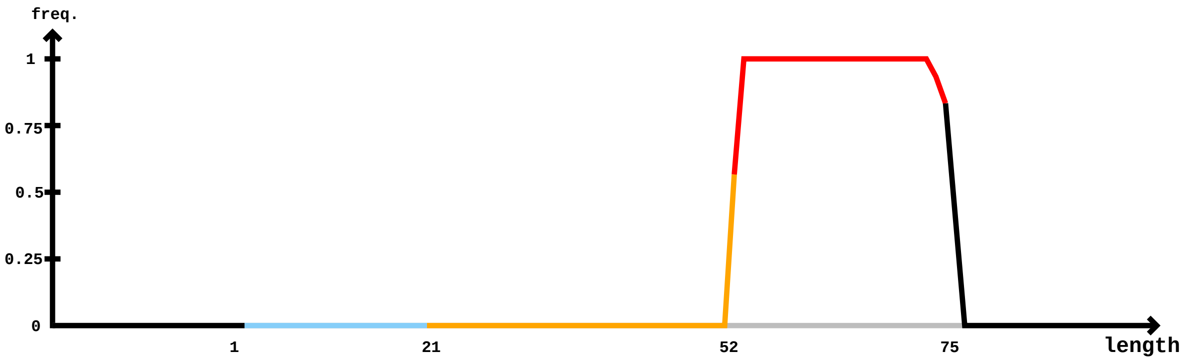


```
                                         Star                                                     Mature
5'- aauaauuaauuuauuuggcugcaccaggucuuagcugcagcacgcaggaucugcgaucuuuguuguggcaugcgggaucuuuagguguggcaugcaggaucuacuucccugac   -3'    exp
    .................(((((((((((((((.((((((((((((((...))))).....)))))))))....)))))))...))))))))...((((.........))))..     reads      mm        sample
    .....................................................................augcgggaucuuuagUuguggca.....................     17         1         seq
    ......................................................................ugcgggaucuuuagCugugg.......................     2          1         seq
    ......................................................................ugcgggaucuuuagCuguggc......................     3          1         seq
    ......................................................................ugcgggaucuuuagCuguggca.....................     7          1         seq
    ......................................................................ugcgggaucuuuagguguggcau....................     1          0         seq
```
